# Reading the Signature of Autophagy in the Ischemic and Infarcted Heart: A Systematic Review of Circulating Biomarkers

**DOI:** 10.3390/ijms27052116

**Published:** 2026-02-24

**Authors:** Davide Radaelli, Asma Alshaeb, Ibrahim Al-Habash, Viktorija Belakaposka Srpanova, Zlatko Jakovski, Gianfranco Sinagra, Anita Galic Mihic, Stefano D’Errico

**Affiliations:** 1 Department of Medical Surgical and Health Sciences, Cattinara University Hospital, University of Trieste, Strada di Fiume, 34149 Trieste, Italy; davide.radaelli@phd.units.it (D.R.); gianfranco.sinagra@asugi.sanita.fvg.it (G.S.); 2 Department of Microbiology, Pathology and Forensic Medicine, Faculty of Medicine, The Hashemite University, Zarqa 13133, Jordan; asma94v.l@hotmail.com; 3 Forensic Medicine Department, Mutah University, Karak 61710, Jordan; ibrhbsh_forensic@yahoo.com; 4 Institute for Forensic Medicine, Criminalistic and Medical Deontology, Medical Faculty, 1000 Skopje, North Macedonia; viktorija.belakaposka@medf.ukim.edu.mk (V.B.S.); drjakovski@gmail.com (Z.J.); 5 School of Medicine, University of Zagreb, Šalata 11, 10000 Zagreb, Croatia

**Keywords:** acute coronary syndrome, autophagy, biomarkers, ischemic heart disease, myocardial infarction

## Abstract

Ischemic heart disease is the main cause of death worldwide. Classic cardiac biomarkers, such as troponin, which are released due to myocyte necrosis, are widely used for diagnosis, but they provide limited information about the initial underlying cellular processes involved in myocardial infarction. Autophagy is now considered fundamental in the pathophysiology of cardiac ischemia and related reperfusion injury. This systematic review aims to identify and highlight candidate autophagy-related biomarkers in cardiac ischemia and infarction with potential benefits for early diagnosis, prognosis, and therapy. A comprehensive literature search was conducted up to 1 June 2025. We included studies that examined biomarkers involved in the autophagy process in cardiac ischemia/infarction, which involved humans and animal models. A total of 14 eligible articles were reviewed. Thirteen autophagy-related biomarkers were identified, including LC3-II/I, Beclin-1, ATG5, ATG7, p62, WIPI1, FGF21, CHRF, Rubicon, IL-1β, IL-18, and adiponectin. These biomarkers have a dynamic pattern, and they exhibited time-dependent changes during the different stages of myocardial infarction. Autophagy biomarkers present a promising understanding of the molecular mechanism of early myocardial ischemia and infarction. Integration of autophagy biomarkers with the classic markers should improve risk stratification, therapeutic decision-making, and prognosis in patients with ischemic heart disease.

## 1. Introduction

Diseases of the cardiovascular system are the top contributors to mortality worldwide. Ischemic heart disease is the most prevalent, accounting for approximately 9 million deaths annually (13% of the total deaths) [1]. Acute myocardial infarction (AMI), which is a myocardial cell death caused by prolonged ischemia due to coronary artery disease, has a high morbidity and mortality [2]. Despite advanced management protocols, including reperfusion therapy, it is still a problematic illness with persistent morbidity and mortality. Thus, we need improvement in risk stratification, early diagnosis, and new therapeutic approaches [3,4].

Autophagy is an essential cellular process responsible for the degradation and reuse of internal components, such as organelles, proteins, and pathogens [5]. Autophagosomes are double-membrane vesicles that sequestrate these components and fuse with lysosomes for the processes of degradation and recycling [6]. Autophagy plays a fundamental role in cellular homeostasis and is upregulated in stressful conditions such as hypoxia [7].

Like other parts of the body, autophagy is important for maintaining normal function in the heart. Abnormalities in autophagy occur in ischemia and infarction [8]. The mechanism by which autophagy plays a role in cardiac ischemia/reperfusion (I/R) injury is complex. Autophagy is normally activated in response to ischemia (adaptive response) to enhance the survival of cardiomyocytes by removing the damaged organelles [9]. But excessive or impaired response can adversely lead to cardiomyocyte damage, death, and cardiac remodeling [10].

Autophagy biomarkers in cardiac ischemia or infarction are essential for novel risk categorization, diagnosis, prognosis, and therapy. Troponins deal with myocyte necrosis once damage is irreversible and are widely used worldwide in the management of cardiac ischemia, but they provide limited information about the earlier pathophysiology of the damaged cardiomyocyte cellular status [11]. The role of autophagy biomarkers could be important in the early pathophysiology, novel risk assessment, and novel therapies.

Despite the widespread knowledge about the importance of autophagy in cardiac ischemia, there are no comprehensive reviews about autophagy-related biomarkers for cardiac ischemia. Other reviews focused on the role of autophagy in cardiac ischemia or other biomarkers related to cardiac ischemia [12,13], but none have specifically focused on biomarkers directly linked to the autophagy process during myocardial ischemia or infarction.

We establish a base for further research into biomarkers that provide more comprehensive information related to cardiac ischemia, including preventive medicine development, clinical implications (diagnosis, management, and prognosis), forensic medicine investigations, and therapeutic strategies.

## 2. Materials and Methods

### 2.1. Eligibility Criteria

In this systematic review, all relevant literature available in the PubMed database from its inception to 1 June 2025 was systematically retrieved. The study included all papers that met the following criteria: original research articles, including both prospective and retrospective designs, reviews, or short communications; published in English; investigated potential molecules or proteins detectable in plasma samples; the molecules studied were related to the autophagy pathway; and involved subjects with confirmed acute myocardial ischemia or infarction, regardless of whether it was a first presentation or in the context of pre-existing coronary artery disease. Studies conducted on both human and animal models, particularly mice models, were involved. Journal Impact Factor was not considered in the eligibility or exclusion of studies; selection was based solely on scientific relevance and predefined methodological criteria.

**Figure 1:**
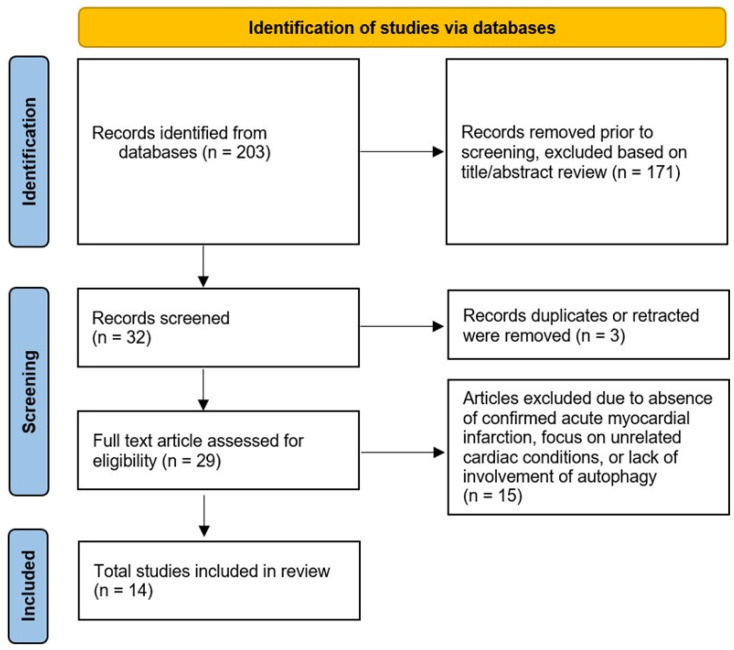
PRISMA 2020 flow-diagram.

The search terms used were “myocardial ischemia”, “myocardial infarction”, “autophagy” and “biomarker” restricted to titles, abstracts, and keywords. Studies that do not involve investigation of such potential biomarkers were excluded from consideration. Each selected paper underwent thorough review and cross-referencing to find other pertinent research. Based on the Reporting Items for Systematic Reviews and Meta-Analyses (PRISMA) 2020 Guidelines [14], a methodological assessment of every study was carried out (Figure 1).

### 2.2. Study Selection

Data collection was conducted independently by two researchers, A.S. and I.H., who separately reviewed the retrieved articles. Using the predefined search strategy, a total of 203 records were identified in the database. After screening the titles and abstracts, 29 articles were selected for full-text reviewing based on their alignment with the research criteria and objectives. Upon further assessment, studies focusing on coronary artery disease (CAD) without confirmed acute myocardial infarction, those addressing unrelated cardiac conditions, or those not involving autophagy were excluded. Ultimately, 14 articles met the inclusion criteria and were included in this review.

### 2.3. Data Extraction

The data extraction was independently carried out by two researchers (A.A. and I.H.), with each reviewer cross-verifying the other’s results for accuracy. When discrepancies arose, the authors (A.G., Z.J., V.B.S., and D.R.) engaged in discussion, ultimately reaching a unified decision. The 14 included articles met our criteria and involved 13 candidate autophagy-related biomarkers. The extracted data were subsequently compiled into a summary table (Table 1).

The following details were gathered for all included articles: the article title, the proposed biomarkers, the involved sample (subject group), the used methods, and the results at the end of the study. The overall dataset was reviewed by a cardiologist (G.S.) and cardio-pathologist (S.D.) to ensure clinical relevance and accuracy.

To facilitate the graphical representation of apoptotic pathways activated in cardiomyocites following ischemic insult, established information derived from the scientific literature was provided to ChatGPT (OpenAI, San Francisco, CA, USA). The AI system was used solely to assist in generating a schematic draft of the explanatory figure. All biological content, pathway and accuracy, and final figure design were critically reviewed, verified and edited by the authors.

## 3. Results

Out of the total 14 studies that met the eligibility criteria and were included in this systematic review, 13 distinct autophagy-related biomarkers were identified and are presented herein.

### 3.1. MAP-LC3 (Microtubule-Associated Protein Light Chain 3) (LC3II/I)

LC3 is considered one of the autophagy-related (ATG) proteins involved in the autophagic process. LC3 is a subunit of microtubule-associated proteins 1A and 1B (MAP1LC3). It is similar to the yeast protein ATG8. All mammalian ATG8 homologues play a role in autophagosome formation in the autophagy process. Two types of LC3 are involved in the process of autophagy: LC3-I (cytosolic form) and LC3-II (membrane-bound form). Upon the process, LC3-I is formed by LC3β. After that, LC3I converts to membrane-bound LC3II via Conjugation with phosphatidylethanolamine (PE). LC3II is considered to be very important for autophagosome formation. Levels of LC3-II proportionally correlate with the number of autophagosomes [28,29]. According to Mo et al. (2021), LC3-II/I notably increases during I/R injury [15]. Kong et al. (2023) also reported increase in LC3-II in acute myocardial infarction before coronary revascularization [17]. In another study, this autophagy marker was reduced 6 months after myocardial infarction (rehabilitation period) [16].

### 3.2. Nuclear Pore Glycoprotein P62

P62, which is also called sequestosome 1 (SQSTM1), is a stress-inducible intracellular protein that regulates different transduction pathways responsible for cell survival and death [30]. It is one of the receptors that are involved in selective autophagy [31]. It is usually found in cellular inclusion bodies with polyubiquitinated proteins and also in cytosolic protein aggregates, which accumulate in some situations, such as some chronic diseases. It is normally degraded by autophagy [32]. This explains the decrease in P62 level noticed by Mo et al. (2021) during I/R injury [15]. Even though autophagy itself can modulate the level of P62, it can inhibit autophagy by activation of mammalian target of rapamycin complex 1 (mTORC1) [30].

### 3.3. ATG7 (Autophagy-Related Gene 7)

A multifaceted core ATG protein that drives the essential stages of autophagy [33]. It could be linked to innate immunity via LC3-associated phagocytosis, unconventional protein secretion, receptor recycling, exocytosis of secretory granules, and modulation of p53-dependent cell cycle arrest and apoptosis [34,35]. ATG7 is upregulated in I/R injury. This can be explained by the role of the Cardiac hypertrophy-related factor (CHRF) effect on the miR-182-5p/ATG7 pathway [36]. In I/R injury, CHRF increases, leading to sequestration of miR-182-5p. ATG7 is upregulated when miR-182-5p is downregulated. This sequence means that CHRF sequestrates miR-182-5p to regulate ATG7 [15].

### 3.4. CHRF

CHRF is a type of lncRNA that regulates multiple cardiac diseases [36,37]. CHRF is upregulated in cases of myocardial I/R injury in vivo and in vitro models. It was found that CHRF aggravates myocardial I/R injury through the aggravation of autophagy in the mean of the modulation of miR-182-5p/ATG7 pathway. Silencing of CHRF causes a decrease in myocardial I/R injury in addition to suppression of autophagy, as noticed by the reversal of the changes occurring for both LC3 and P62 [15].

### 3.5. NLRP3 (NLR Family Pyrin Domain Containing 3)

NLRP3 is a sensor located intracellularly to detect abnormal dangerous signaling [38]. Disturbances in cellular hemostasis are sensed by NLRP3, which lead to activation of caspase-1, interleukin 1 beta (IL-1β), and Interleukin-18 (IL-18) [39,40]. In cases of myocardial ischemia and infarction, activation of NLRP3 inflammasome leads to further damage of cardiac muscle through direct promotion of cell death and indirect initiation of inflammation in the cardiac muscle mediated by interleukins (IL-1β, IL-18) [40]. The NLRP3 inflammasome is inhibited by autophagy, but the exact mechanism is still unclear [41]. Autophagy impairment in cases of AMI, noticed by lower levels of MAP-LC3, could explain this increase in NLRP3 during AMI [16].

### 3.6. IL-1β and IL-18

Both interleukins are activated by the inflammasome, which is a protein complex including Nod-like receptor (NLR), the precursor procaspase-1, and the adaptor ASC (apoptosis-associated speck-like protein containing a CARD). This inflammation causes harmful effects on the heart, and the interruption of this activation is considered to be a protective measure for the heart [42,43]. In cases of AMI, these interleukins increase in response to the effect of NLRP3 inflammasome. Impairment of autophagy leads to an exaggerated effect of these inflammatory interleukins on the cardiac muscle [16].

### 3.7. ATG5 (Autophagy-Related Gene 5) and Beclin 1

ATG5 and Beclin 1 are both essential autophagy regulators that are involved in the formation of the autophagosome. Both have a central role in autophagy, so their disruption or dysregulation may result in impairment of autophagy and may be associated with several pathological conditions [10,44,45,46]. Independent of autophagy, ATG5 is involved in other functions, including control of mitochondrial quality following oxidative damage, immune-related functions, adipocyte differentiation, and apoptosis [47,48]. Beclin 1 acts primarily through its interaction with class III-type phosphoinositide 3-kinase (class III PI3K). In response to various stress signals, Beclin 1 contributes to cardiomyocyte survival by promoting autophagic flux and limiting cell death during I/R injury [10,49].

Kong, Min Gyu, et al.’s study showed that the level of ATG5 and Beclin 1 was significantly elevated in the serum of patients with AMI before revascularization [17], and in the study by Grazide et al., these levels were lower compared to individuals without CAD in the late phase of reperfusion [18]. This initial upregulation followed by downregulation can be explained by the change in the autophagy flux; it is stimulated once the cells are exposed to ischemic injury and its associated oxidative stress and hypoxia, leading to elevated levels of both regulators. Then, with restoration of perfusion, particularly after enough time of reperfusion, there will be the inactivation of autophagy flux, which explains the low level. Furthermore, Beclin 1 has been shown to be elevated even in the early reperfusion triggered by reactive oxygen species formation in the I/R stage, which promotes excessive autophagy and induces further injury [50,51].

### 3.8. FGF21 (Fibroblast Growth Factor 21)

FGF21 is a cardioprotective hormone expressed by various tissues in the body, working as an endocrine hormone primarily to correct metabolic dysfunction related to energy and glucose homeostasis [52,53]. It is mainly expressed by the liver, with smaller contributions from the heart, muscle, brain, pancreas, and adipose tissue [54,55]. Its expression is induced in response to stressful conditions, such as ischemia-induced injury, exercise, and nutritional stresses [56,57]. It inhibits cell apoptosis, up-regulates the expression of antioxidants, and suppresses endoplasmic reticulum stress, all of which contribute, in myocardial infarction (MI), to preventing cardiomyocyte death and helping them survive during prolonged ischemia [58,59,60].

In addition, it is considered one of the autophagy-related proteins whose activity is mediated through signaling molecules such as Beclin1, protein kinase B (AKT), transcription factor EB (TFEB), LC3, and 5-adenosine monophosphate-activated protein kinase (AMPK) [61,62,63]. As a result, it helps protect cardiac cells from ischemic injury and promotes autophagic flux to protect cardiomyocytes during I/R [64].

In patients with CAD, the production and release of cardiac FGF21 has been observed to increase [19,20,21]. Clinical studies found that the circulating FGF21 levels were elevated in all CAD subtypes, AMI, stable angina pectoris (SAP), and unstable angina pectoris (UAP), peaking during the first day of infarction, and the high level remained for several days after the infarction. Notably, patients with higher FGF21 levels one week after infarction experienced reinfarction within a month, and this higher level is associated with a high mortality rate [19]. Also, the study by Cheng et al. illustrates a correlation between elevated FGF21 and cardiac troponin I as well as Creatine Kinase-MB (CK-MB) levels [21], while another study by Sunaga et al. found that FGF21 levels correlated with adipose tissue lipolysis byproducts such as fatty acid binding protein 4 (FABP4) and saturated fatty acids, but not with cardiac troponin T [20]. However, the high level is not related to the age, sex, body weight, or glucose levels [19]. These findings highlight its potential as an independent and strong biomarker.

### 3.9. APN (Adiponectin)

APN is an adipocyte-derived adipokine with well-known anti-inflammatory and cardioprotective effects. It plays an important role in various physiological processes, including energy metabolism, insulin sensitivity, and vascular homeostasis [65,66]. Several studies have demonstrated that APN has a cardioprotective role, as it has an essential role in regulating myocardial autophagy. This function of APN is mediated primarily through the activation of AMPK and p38 mitogen-activated protein kinase (MAPK) signaling pathways [67].

Autophagy appears to be impaired in the absence of APN, so its deficiency is associated with abnormal cardiac function [68,69,70]. For instance, an experimental study using APN-deficient mouse models subjected to MI further confirmed the impaired autophagic response, resulting in exacerbated cardiac damage [71].

Herein, three studies have linked circulating APN levels with AMI. It has been observed that plasma APN concentrations are significantly lower in those with AMI compared to controls, suggesting it as a biomarker for myocardial injury [67].

### 3.10. WIPI1 (WD Repeat Domain Phosphoinositide-Interacting Protein 1)

WIPI1 is one of the autophagy regulators; specifically, it is involved in mitophagy, which is a specialized form that deals with mitochondrial integrity maintenance and reduces oxidative stress [72]. Its autophagy function plays a role in immunological responses, cancers, and infections [72,73]. Furthermore, it is involved in cardiovascular diseases, particularly ventricular failure when it is upregulated [74], and diabetic cardiomyopathy with its reduction [72]. It has been explained that its upregulation causes autophagy promotion, while its downregulation is associated with mitophagy impairment, worsening cardiac function.

Notably, WIPI1 is significantly elevated within one hour of AMI, explained by the increased making it a promising early diagnostic biomarker. This upregulation was explained as probably reducing the infarction progress by activating autophagy pathways [26].

### 3.11. Rubicon

Rubicon is another regulator of autophagy but has a negative role that inhibits the maturation of autophagosomes by interacting with the Beclin 1-UVRAG-Vps34 complex and suppressing Rab7 activity [75,76]. However, Rubicon is essential for LC3-associated phagocytosis (LAP) and endocytosis (LANDO), as it promotes immune regulation and lysosomal degradation [76,77]. In MI, Rubicon is upregulated during I/R injury, contributing to impaired autophagic flux and increasing the cardiac injury. Its dual role in suppressing autophagy while supporting LAP may disrupt cellular balance under stress. Thus, Rubicon is another potential biomarker in MI. Additionally, it was demonstrated that its level is related to both total and low-density lipoprotein (LDL) cholesterol levels, but the Rubicon level generally is independently related to the risk of infarction [27].

## 4. Discussion

Autophagy is a tightly regulated intracellular degradation pathway essential for maintaining cellular homeostasis and quality control under basal conditions. It is responsible for removing damaged organelles, misfolded proteins, and unwanted cytoplasmic components via lysosomal degradation [78]. Under stress conditions such as hypoxia, nutrient deficiency, and oxidative stress, autophagy is markedly upregulated [6,79]. Physiologically, autophagy regulates many processes such as aging, embryogenesis, immunity, and cell differentiation [5,80]. Abnormal autophagy contributes to many pathological conditions such as neurodegenerative disorders, cardiovascular diseases, infections, and cancer [81,82].

In the context of AMI, the autophagic response is neither uniform nor unidirectional; rather, it oscillates between cytoprotection and cytotoxicity depending on the timing, extent, and molecular regulation [9,83,84,85].

**Figure 2:**
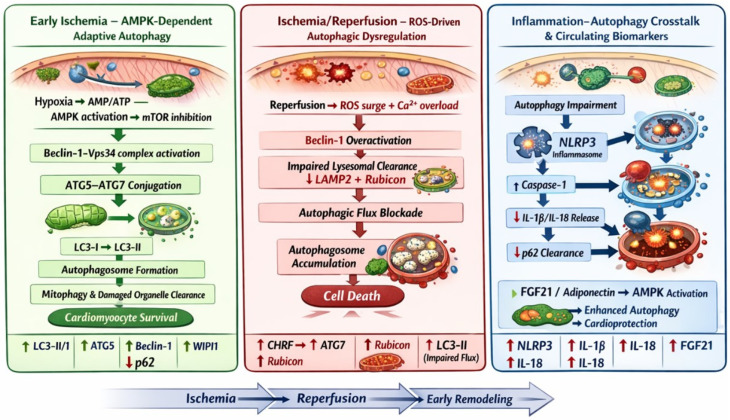
Autophagic response in cardiomyocites during ischemia and ischemia/reperfusion injury. Early ischemia activates AMPK and inhibits mTOR, promoting Beclin-1-Vps34 signaling, ATG5-ATG7 conjugation, LC£-I to LC3-II conversion, and autophagosome formation. In this adaptive phase, autophagy supports mitophagy and cardiomyocyte survival. During reperfusion, ROS generation and Ca^2+^ overload induce Beclin-1 overactivation and impaired lysosomal clearance, resulting in defective autophagic flux and autophagosome accumulation, contributing to cell death. Autophagy impairment enhances NLRP3 inflammasome activation and IL-1β/IL-18 release, while FGF21 and adiponectin modulate AMPK-dependent cardioprotective responses. These dynamic changes support the diagnostic and prognostic relevance of autophagy-related biomarkers in acute myocardial infarction.

This systematic review presents several potential biomarkers involved in the process of autophagy useful in the myocardial acute phase or during the phase of I/R injury, highlighting their diagnostic, prognostic, and therapeutic potential. Some of those candidate biomarkers are direct indicators of autophagy (involved in autophagosome formation, maturation, elongation, fusion, and degradation), such as LC3II/I, P62, ATG7, ATG5, Beclin 1, WIPI1, and Rubicon; others are indirect indicators, like CHRF, NLRP3, IL-1β, IL-18, FGF21, and APN, that regulate the process either by promoting or suppressing it according to the specific function and condition (Figure 2).

The findings illustrate that the alterations in the levels of the proposed biomarkers are time-dependent. During the early phase of AMI, several biomarkers, such as WIPI1, NLRP3, IL-1β, and IL-18, were found to be elevated in circulation. In contrast, APN showed significantly lower levels in the early phase of MI, and other candidate biomarkers, including CHRF, ATG7, and Rubicon, showed elevated levels during the I/R phase rather than the initial onset of MI. At this same phase, P62 levels were reduced. Some of the biomarkers, such as ATG5 and Beclin-1, followed a biphasic pattern; their levels were significantly elevated in the early infarction stage but subsequently declined below control levels during the late reperfusion phase. Also, another biomarker, LC3-II/I, displayed a biphasic pattern; its levels were higher in MI patients compared to controls during both the early infarction and the I/R stage, yet they declined markedly during rehabilitation. Additionally, FGF21 demonstrated a sustained elevation, peaking during early infarction and remaining high throughout the I/R period.

Out of those proposed biomarkers, WIPI1 exhibited a unique feature in that it was detected to rise within one hour of the infarction, highlighting its diagnostic role in the earliest time, especially when combined with traditional markers like troponins, even if the symptoms are not severe enough yet [26]. Regarding the prognostic features, APN, which was downregulated in the circulation during AMI, had its high circulating level linked with lowering MI risk in a prospective study that followed up men for 6 years [86]. Another one with a prognostic feature is FGF21, as its high level after a week of the infarction suggests that this patient is at a high risk to experience reinfarction within 30 days [19].

Furthermore, the change exhibited by the alteration of the CHRF, ATG7, and Rubicon levels during only one stage can be useful as a diagnostic tool and an indicator; upon its elevation, it indicates the ischemic reperfusion injury is starting, giving a time spot that may be beneficial for many therapies directed to minimize these harmful period consequences [87,88,89,90].

The harmful effect of autophagy in I/R was also reported through studying the axis of CHRF, miR-182-5p, and ATG7, which was discussed by Mo et al. (2021). CHRF, an lncRNA, is upregulated during I/R [36,37]. This upregulation causes an increase in the risk of myocardial injury. Silencing CHRF enhances cellular viability and decreases infarct size, lactate dehydrogenase (LDH) activity, and apoptosis in cardiomyocytes exposed to I/R. CHRF sequestrates miR-182-5p, preventing it from inhibiting ATG7 (a key gene for autophagy). ATG7 decreased, leading to autophagy attenuation when miR-182-5p upregulated in response to CHRF suppression. This is supported by the decreased level of LC3-II/I and the restoration of P62 expression level in response to CHRF suppression, which indeed indicates decreased autophagy activity. This suggests that CHRF increases autophagy activity in stress conditions, as both LC3-II/I upregulation and P62 degradation reflect the autophagy activity. According to that, CHRF is suggested to exacerbate myocardial I/R injury by activation of autophagy. Modulation in the axis of CHRF, miR-182-5p, and ATG7 could be helpful in the therapeutic strategies concerned in this field [15].

Only one paper discussed the role of autophagy in the post-MI rehabilitation period. According to [16], a significant decrease in the level of MAP-LC3 in rehabilitated MI patients compared to controls was noticed, indicating a decrease in autophagosome formation. In contrast, NLRP3 inflammasome and IL-1β levels were elevated, suggesting that the activation of an inflammatory status leads to defective autophagy activity. This defect could affect the clearance of the unwanted cellular components and stimulate the inflammatory response, which affects repair and recovery. Given these findings, monitoring of LC3 and inflammasome activity could be very helpful for rehabilitation quality improvement. Also, focusing on increasing the expression of LC3 in rehabilitated MI patients could inhibit the inflammatory response and promote the repair and recovery of myocardium [16].

Kong et al. [17] investigated the changes in autophagy biomarkers in AMI patients and compared them with the usually used cardiac biomarkers and clinical parameters. The study revealed a correlation between the elevated autophagy markers, LC3-II and Beclin 1 in the coronary artery, and cardiac Troponin T and NTproBNP levels, which indicates the presence of a relationship between autophagy activity and cardiac injury severity. In addition, there was no association between autophagy markers and ventricular ejection fraction, except for WIPI1, which was found to be elevated in ventricular failure, attributing the phenomenon to its role in promoting autophagy, and demonstrated reduced levels in diabetic patients who developed diabetic cardiomyopathy, as its reduction impairs mitophagy, resulting in deterioration of cardiac function [72,74]. Additionally, a negative correlation has been reported in one of the involved articles between APN levels and C-reactive protein (CRP) in AMI patients, suggesting that the decrease in APN is not only a marker of infarction but may also reflect the degree of systemic inflammation associated with it [25]. According to that, integrating autophagy markers with the traditional cardiac biomarkers could be beneficial in investigating the extent of cardiac damage and indeed in improving therapeutic strategies [17].

Despite significant advances, substantial gaps remain in the explanation and the exact role of autophagy in cardiac ischemia and infarction. The temporal dynamics of autophagy-related biomarkers, prior to ischemic insult, during ischemia, throughout I/R, and in the post-infarction period, are not yet fully elucidated. Moreover, it remains unclear whether these alterations in autophagy markers are involved in the causative pathological status in myocardial injury or are secondary responses to ischemic stress.

Reperfusion-related ventricular arrhythmias, especially ventricular fibrillation, are a well-recognized cause of sudden cardiac death in both laboratory models and patients undergoing myocardial reperfusion [91,92]. Although restoring coronary blood flow is necessary to limit infarct size, the process itself can inversely affect the myocardium. The sudden return of oxygen and substrates is accompanied by intracellular calcium accumulation, mitochondrial injury, and a surge in reactive oxygen species. These alterations are closely associated with cell death pathways, including necrosis and autophagy [8,93].

Autophagy in this setting appears to have context-dependent effects. Basal activity may support cellular adaptation; however, excessive activation or interruption of autophagic flux during reperfusion can intensify mitochondrial dysfunction, reduce ATP availability, and disrupt ionic gradients. Such metabolic and structural disturbances can alter membrane excitability and conduction, creating conditions favorable for malignant ventricular arrhythmias [8,85,94,95].

Clinical data that directly link autophagy markers to reperfusion arrhythmias are still limited. Clarifying this relationship may help to define whether targeting autophagic pathways may have potential in reducing lethal reperfusion-induced arrhythmias.

Another considerable challenge regarding the death of cardiomyocytes during myocardial ischemia and reperfusion, as an example of cellular death in general, is that it normally occurs as a complex set of processes, not through isolated pathways. These processes mainly include autophagy, apoptosis, and necrosis, which usually coexist and interact [96]. Increasing evidence indicates that autophagic, apoptotic, and necrotic pathways converge at common regulatory nodes, including p53, Bcl-2 family proteins, and RIP kinases, highlighting the integrated nature of cellular death signaling [97]. Autophagy may initially start as a cytoprotective mechanism in response to stressful stimuli. However, excessive or dysregulated autophagy can facilitate apoptotic or necrotic cell death [98,99]. Several molecules, which could be considered as biomarkers, including Beclin-1, ATG5, LC3, p62, and caspases, can all be affected during the whole process through the three mechanisms, which indeed highlights the overlapping of these processes regarding cellular death [100,101,102]. For instance, cleavage of autophagy-related proteins such as Beclin-1 and ATG5 by caspases shifts the process of autophagy toward apoptosis [103,104], and modulation of RIPK1/RIPK3 or MLKL can direct cells toward necrotic-like death even when autophagy or apoptosis is active [105]. It is important to recognize this overlap when interpreting the related biomarkers, as changes in autophagy-related markers may reflect not only autophagic activity but also broader cell death dynamics occurring within the ischemic myocardium.

The contribution of autophagy and its associated biomarkers to the subsequent outcomes, including recurrent ischemic events and the development of heart failure, also remains underexplored. Additionally, another challenge in this field is the absence of standardized methodologies for detecting and quantifying autophagy markers, which hinders inter-study comparability and the development of clinically valid conclusions.

The significant results about autophagy markers in different aspects regarding cardiac infarction encourage further studies. Further research is needed for validation before clinical implementation in order to comprehensively improve diagnosis, prognosis, risk stratification, therapeutic strategies, and rehabilitation protocols.

## 5. Conclusions

This systematic review aims to identify and highlight the potential autophagy-related biomarkers in cardiac ischemia and infarction cases. Autophagy markers may help improve protocols and strategies related to ischemic heart disease, including diagnosis, prognosis, and management. Several markers, such as LC3-II/I, ATG5, Beclin 1, FGF21, and WIPI1, could be beneficial in determining high-risk individuals, identifying the stages of the disease, modifying the pathway of management, and predicting the outcome. For a clinical application, further validation and standardization are needed.

## Figures and Tables

**Table 1:** Overview of Included Studies Reporting Autophagy Biomarkers Implicated in Acute Myocardial Infarction.

	Articles	Biomarker	Sample Source	Tissue Type	Methods	Results
1	Mo, Yipeng et al. [15]	CHRF	Male Sprague–Dawley rats (*n* = 6 per group, total 5 groups)	Myocardial tissue	RT-qPCR, RNA pull-down, luciferase assay, TUNEL, TTC staining, MTT assay	Upregulation of CHRF in ischemia–reperfusion injury (I/R). Silencing CHRF leads to decrease in the size of infarct, LDH, apoptosis, and autophagy markers, which indicate a protective effect.
		ATG7	H9C2 rat	Cardiomyoblast cells	RT-qPCR, Western blot, luciferase assay	ATG7 is a direct target of miR-182-5p. CHRF indirectly increases ATG7. Overexpression of ATG7 restores autophagy even when CHRF is silenced.
		LC3-II/I, p62	H9C2 rat	Cardiomyoblast cells	Western blot, GFP-LC3 immunofluorescence	LC3-II/I is increased and p62 decreased under I/R (indicating enhanced autophagy). These changes are reversed by CHRF knockdown.
2	Bullón, Pedro, et al. [16]	NLRP3	H9C2 rat	Cardiomyoblast cells	qPCR (SYBR Green)	Higher mRNA expression in AMI patients compared to controls. Indicates inflammation and autophagy impairment in cardiac ischemia.
		MAP-LC3	AMI patients 6 months after the attack (*n* = 150) and controls (109 healthy men)	Blood mononuclear cells	qPCR (SYBR Green)	Lower gene expression in AMI group, suggesting reduced autophagic activity.
		IL-1β	AMI patients 6 months after the attack (*n* = 150) and controls (109 healthy men)	Blood mononuclear cells	ELISA	Elevated IL-1β in AMI patients; correlated with inflammasome activation and cardiovascular risk.
3	Kong, Min Gyu, et al. [17]	- LC3-II - ATG5 - Beclin-1	AMI patients 6 months after the attack (*n* = 150) and controls (109 healthy men)	Blood mononuclear cells	ELISA	LC3-II, ATG5, and Beclin-1 levels are significantly higher in AMI patients.
4	Grazide et al. [18]	ATG5 and Beclin 1	22 AMI patients, 19 controls	Human serum, peripheral artery and coronary artery before coronary revascularization	ELISA	The circulating concentrations of ATG5 and Beclin 1 were lower in AMI patients compared with control subject.
5	Ma et al. [10]	Beclin 1	AMI patients (*n* = 100) and control subjects (*n* = 99) at high cardiovascular risk but without known coronary disease.	Blood	Immunoblots	Ischemia/reperfusion injury impairs autophagosome clearance mediated in part by reactive oxygen species–induced decline in lysosome-associated membrane protein-2 and upregulation of Beclin 1, contributing to increased cardiomyocyte death.
6	Zhang et al. [19]	FGF21	Adult male cardiomyocyte-specific green fluorescent protein–light chain-3 (GFP-LC3) transgenic mice and C57BL/6 mice.	Myocardial tissue	ELISA	FGF21 levels reached the maximum within approximately 24 h after the onset of AMI and remained high on the 3rd and 7th days
7	Sunaga et al. [20]	FGF21	Samples collected from 55 AMI patients and 45 non-AMI control patients on the 1st day. All patients were followed-up within 30 days.	Blood	Western blot analysis and ELISA	Serum FGF21 levels are rapidly elevated upon onset of AMI. AMI patients had higher serum FGF21 levels on admission than SAP.
8	Cheng et al. [21]	FGF21	A total of 93 patients, 43 were diagnosed as SAP, and 50 were as AMI. Also, experiments using mice model of myocardial ischemia were used.	Blood	ELISA	FGF21 levels on admission were significantly higher in UAP patients than controls, and also higher than SAP patients.
9	Sinha et al. [22]	FGF21	A total of 197 subjects were categorized into: 66 subjects with SAP, 76 subjects with UAP, 55 control subjects with normal coronary artery findings	Blood	ELISA	The case group had considerably higher levels of FGF21 compared to the control group.
10	Shibata et al. [23]	Adiponectin	The study enrolled a total of 110 subjects, of which 70 were in case groups (patients with SAP having atherosclerotic lesions on angiography) and 40 were in the control group.	Blood	Plasma adiponectin by ELISA. Heart adiponectin: by immunohistochemical, Western blot and real-time PCR analyses.	Adiponectin accumulates in the heart following ischemic damage primarily through leakage from the vascular compartment, and that adiponectin has a longer half-life in damaged heart tissue than in plasma
11	Kumada et al. [24]	Adiponectin	Wild-type (WT) and adiponectin-knockout (APN-KO) mice	Plasma, Myocardial tissue	ELISA	Male patients with hypoadiponectinemia had a significant 2-fold increase in CAD prevalence, independent of well-known CAD risk factors.
12	Kojima et al. [25]	Adiponectin	225 male patients were enrolled from inpatients who underwent coronary angiography. Voluntary blood donors (*n* = 225) matched for age served as controls.	Blood	ELISA	Plasma adiponectin concentrations in the patients with AMI on admission were significantly lower than those in the control subjects.
13	Liu et al. [26]	WIPI1	34 patients with AMI, and the control group consisted of 35 subjects who had atypical chest pain at rest, or with some exercise, but had no significant coronary artery stenosis (<25% of luminal diameter) and no coronary spasm.	Blood	Polymerase chain reaction and Western blots	Protein WIPI1 specifically expressed in heart was significantly increased within an hour after AMI.
14	Grazide et al. [27]	Rubicon	Thirty male mice (8 weeks old) were randomly divided into different experimental groups	Myocardial tissue	ELISA	Significantly elevated plasma Rubicon levels in AMI patients compared to the control group Multivariate analysis confirmed that Rubicon levels were independently associated with an increased risk of MI.

CHRF: Cardiac Hypertrophy–Related Factor; ATG7; autophagy-related gene; TTC: Triphenyltetrazolium Chloride; TUNEL: Terminal Deoxynucleotidyl Transferase dUTP Nick End Labeling; MTT: 3-(4,5-dimethylthiazol-2-yl)-2,5-diphenyltetrazolium bromide assay; LDH: Lactate Dehydrogenase; qPCR: Quantitative Polymerase Chain Reaction; NLRP3: NLR family pyrin domain containing 3; AMI: Acute Myocardial Infarction; MAP-LC3: 1-Microtubule-associated protein light chain 3; IL-1β: Interleukin-1 beta; ELISA: Enzyme-Linked Immunosorbent Assay; ATG5: Autophagy-related gene 5; FGF21: Fibroblast growth factor 21; SAP: stable angina pectoris; UAP: unstable angina pectoris; CAD: Coronary artery disease; WIPI1: WD repeat domain phosphoinositide-interacting protein 1.

## Data Availability

The original contributions presented in this study are included in the article. Further inquiries can be directed to the corresponding authors.

## Abbreviations

The following abbreviations are used in this manuscript:

AMI	Acute Myocardial Infarction
APN	Adiponectin
ATG	Autophagy-Related Gene
CHRF	Cardiac Hypertrophy–Related Factor
ELISA	Enzyme-Linked Immunosorbent Assay
FGF21	Fibroblast Growth Factor 21
I/R	Ischemia/Reperfusion
IL-1β	Interleukin-1 beta
IL-18	Interleukin 18
LC3	Light Chain 3
MAP-LC3	Microtubule-Associated Protein Light Chain 3
WIPI1	WD Repeat Domain Phosphoinositide-Interacting Protein 1
